# Differences of in vitro immune responses between patent and pre-patent *Litomosoides sigmodontis*–infected mice are independent of the filarial antigenic stimulus used

**DOI:** 10.1007/s00436-024-08365-0

**Published:** 2024-10-22

**Authors:** Kathrin Arndts, Anna Wiszniewsky, Anna-Lena Neumann, Katharina Wiszniewsky, Gnatoulma Katawa, Achim Hoerauf, Laura E. Layland-Heni, Manuel Ritter, Marc P. Hübner

**Affiliations:** 1 Institute for Medical Microbiology, Immunology and Parasitology (IMMIP), University Hospital Bonn (UKB), Venusberg-Campus 1, 53127 Bonn, Germany; 2 German-West African Centre for Global Health and Pandemic Prevention (G-WAC), Partner Site Bonn, Bonn, Germany; 3 Unité de Recherche en Immunologie Et Immunomodulation (UR2IM), Université de Lomé, Ecole Supérieure Des Techniques Biologiques Et Alimentaires (ESTBA), Lomé, Togo; 4 German Center for Infection Research (DZIF), Partner Site Bonn-Cologne, Bonn, Germany

**Keywords:** *Litomosoides sigmodontis*, Antigenic extract, Patent, Pre-patent, Ultracentrifugation, T cell assay

## Abstract

Lymphatic filariasis and onchocerciasis are neglected tropical diseases and cause significant public health problems in endemic countries, especially in sub-Saharan Africa. Since the human parasites are not viable in immune-competent mice, animal models have been developed, among them *Litomosoides sigmodontis* which permits a complete life cycle in BALB/c mice, including the development of patent infections with circulating microfilariae (Mf, the worm’s offspring). To investigate the immunomodulatory properties of helminths in vitro, antigenic extracts can be prepared from different life cycle stages of the *L. sigmodontis* model, including adult worms, but the methods to prepare these antigens differ between research groups. This study analyzed whether different centrifugation methods during the preparation of an antigenic extract, the gender of used worms, or the different fractions (soluble or pellet) altered filarial-specific CD4^+^ T cell responses. These cells were isolated from pre-patent or patent/chronic infected mice, hence those without and those with Mf, respectively. Ex vivo immune responses were compared at these two different time points of the infection as well as the parasitic parameters. Worm burden and cell infiltration were elevated in the thoracic cavity (TC) and draining mediastinal lymph nodes at the pre-patent stage. Within the TC, eosinophils were significantly up-regulated at the earlier time point of infection which was further reflected by the eosinophil-related eotaxin-1 levels. Regarding the production of cytokines by re-stimulated CD4^+^ T cells in the presence of different antigen preparations, cytokine levels were comparable for all used extracts. Our data show that immune responses differ between pre-patent and patent filarial infection, but not in response to the different antigenic extracts themselves.

**Supplementary Information:**

The online version contains supplementary material available at 10.1007/s00436-024-08365-0.

## Introduction

Approximately 200 million people worldwide suffer from filarial infections including lymphatic filariasis (LF), onchocerciasis, loiasis, and mansonellosis (Ehrens et al. 2022; Mediannikov and Ranque 2018; Ramaiah and Ottesen 2014). These infections are caused by the bite of infected insect vectors that transmit infective third-stage larvae of the filariae with their blood meal to the definitive host. Since adult filarial worms have developed mechanisms to evade the host’s immune responses, they can survive for many years (Hoerauf et al. 2011, 2005). The majority of filariae-infected individuals present a homeostatic, regulated immune state (characterized by elevated IL-10, TGF-β, and IgG4 levels and increased numbers in regulatory T and B cells), which is accompanied by high numbers of worms and microfilariae (Mf), the filarial offspring, that is taken up by the insect vector and required for transmission (Adjobimey and Hoerauf 2010; Arndts et al. 2012; Arndts et al. 2015; Babu and Nutman 2014; Fischer et al. 2023; Katawa et al. 2015; Ritter et al. 2018, 2019; Simonsen and Mwakitalu 2013). Nevertheless, some of the infected humans develop severe forms of disease-related symptoms whose underlying mechanisms for these clinical scenarios remain unresolved (Arndts et al. 2012; Arndts et al. 2015; Arndts et al. 2014; Fischer et al. 2023; Katawa et al. 2015; Pfarr et al. 2009). Onchocerciasis and LF are the most important filarial infections in terms of public health impact (Fischer et al. 2023). Besides individuals with pathology (presenting river blindness and dermatitis in case of onchocerciasis, elephantiasis, and hydrocele in case of LF), individuals may also show different states of the infection: In *Onchocerca volvulus* endemic areas, individuals are found to have nodules with adult worms and do not display severe pathology, and they may also possess Mf or not (Arndts et al. 2014). For LF, around 50% of asymptomatic *Wuchereria bancrofti*–infected individuals become patently infected, thus presenting Mf in their blood (Turner et al. 1993). The others remain latently infected (Mf negative), and since these individuals often show no obvious signs of pathology, this cohort remained unstudied for many years. The reasons behind this phenomenon are unclear, but our previous studies showed that the immunological profiles of these two cohorts are quite distinct since elevated adaptive immune responses were seen in latent individuals (Arndts et al. 2012). However, in human infections, it is not easy to differentiate a true latent state from a pre-patent infection (Arndts et al. 2012; Dunyo et al. 2000; Rodrigo et al. 2016). It is very difficult to maintain human filariae in a murine model and, so far, only possible with immunocompromised mice (Chunda et al. 2020; Hong et al. 2019; Patton et al. 2018; Pionnier et al. 2019). Thus, the rodent filarial species *Litomosoides sigmodontis* is often used as a surrogate model to investigate immune responses during filarial infection (Risch et al. 2021), as it enables the complete life cycle, including the release of Mf after ~ 50 days of infection in BALB/c mice. In contrast, infections in C57BL/6 mice are cleared after the development of adult worms around 40 days after infection (Hoffmann et al. 2001; Hübner et al. 2009b; Layland et al. 2015; Le Goff et al. 2002; Petit et al. 1992; Reichwald et al. 2022; Rodrigo et al. 2016; Wiszniewsky et al. 2019). This model therefore provides a platform to decipher immunological mechanisms and pathways at distinct stages of infection. Moreover, it reflects the human infection scenario as only 40–60% of mice become patent (Petit et al. 1992; Rodrigo et al. 2016). Our previous studies showed that filarial-specific CD4^+^ T-cell responses from Mf + mice produced significantly higher amounts of cytokines such as IL-5 and IL-10 than those from Mf − mice (Rodrigo et al. 2016). This expands on a plethora of data showing that control of Mf and worm burden depends on both innate and adaptive immune responses that interact to mediate helminth control (Karunakaran et al. 2023).

Previously, murine studies investigating the immunomodulatory properties of helminth infections were using antigenic extracts for their in vitro experiments. However, the preparation methods of these antigenic extracts differ between research groups (Babayan et al. 2003; Berbudi et al. 2016; Buerfent et al. 2019; Hartmann et al. 2011; Hübner et al. 2009b; Karunakaran et al. 2023; Le Goff et al. 2000; Rodrigo et al. 2016; Torrero et al. 2010) which might alter the outcome of the experiments. In general, soluble helminth extracts are prepared after homogenizing adult worms of both genders on ice, followed by a centrifugation step. Little research has focused on how CD4^+^ T cells respond when they are cultured with either soluble or pellet fractions or if the antigen fractions resulted from an ultracentrifugation (UC) step which might remove cuticula particles from the supernatant.

Within this study, two aspects of filarial infections were investigated. First, it was analyzed if immune responses differ between the pre-patent and the chronic patent stage mice (Rodrigo et al. 2016) in regards to cellular infiltration and chemokine levels at the site of infection (thoracic cavity (TC)). On the other hand, filarial-specific CD4^+^ T cell responses from *L. sigmodontis*–infected BALB/c mice (prepared from the aforementioned two time points of the infection) were determined following stimulation ex vivo with different *L. sigmodontis* antigenic extracts (soluble or pellet worm fractions following standardized centrifugation or UC) prepared from adult worms either of both or individual genders.

## Materials and methods

### Animals and *Litomosoides sigmodontis* infection

This animal study was approved by the local government authorities: Landesamt für Natur, Umwelt und Verbraucherschutz NRW, Germany. All wildtype mice (*Mus musculus*) on the BALB/c background, jirds (*Meriones unguiculatus*), and cotton rats (*Sigmodon hispidus*) were maintained at the Institute for Medical Microbiology, Immunology and Parasitology (IMMIP), University Hospital Bonn (UKB), Germany (AZ87-51.04.2011.A025/01). Animals were purchased from Janvier, France, and were kept in individually ventilated cages in accordance with the Deutsche Tierschutzgesetz (German animal protection laws) and the EU guidelines 2010/63/E4. Infections with *L. sigmodontis* were performed using a natural method as described earlier (Hübner et al. 2009b). In short, intermediate mite hosts (*Ornithonyssus bacoti*) were fed on infected cotton rats with > 1000 Mf/µl of blood. After 10 days, the infected mites were allowed to feed on 6–8-week-old female BALB/c mice or jirds. Thereafter, infective L3 larvae enter the blood and migrate to the TC by days 2–8 p.i. Here, they molt into L4 stage larvae by days 8–12 p.i., become adults by days 28–35 p.i., and start to produce Mf that circulate in the blood by day 50 p.i. (Torrero et al. 2010). BALB/c mice (*n* = 10/infection experiment) were infected with the same batch of mites to compare results at both time points.

### Antigen preparations from patent *L. sigmodontis* worms

For the preparation of *L. sigmodontis* antigenic extracts (LsAg), all procedures were performed under sterile conditions and on ice. Adult worms were isolated from the TC of infected jirds (d260 p.i.), transferred into sterile Petri dishes (Greiner GmbH, Frickenhausen, Germany), and rinsed several times with endotoxin-free sterile PBS (PAA, Linz, Austria). Worms were divided into three groups containing female, male, or a mixture of both and minced for 20 min with a glass potter (VWR, Langenfeld, Germany) until a homogenized solution was achieved. The resulting worm preparations from the female, male, and pooled worm groups were then separated into two different setups. One half was used for the standard centrifugation (STDC) preparation process: centrifugation at 1485 g for 10 min at 4 °C. Afterwards, the supernatant (SN) was transferred into sterile tubes, and the pellet was re-suspended in 1 ml endotoxin-free sterile PBS. The other half was used for the ultracentrifugation (UC) process in which material was transferred into 10 ml UC tubes (Nalgene, New York, USA) and centrifuged at 50,000 × *g* for 2 h at 4 °C. Afterwards, the soluble material was transferred into new sterile tubes, and the pellet was re-suspended in 1 ml endotoxin-free sterile PBS. Protein concentrations of all antigens (female soluble, female pellet, male soluble, male pellet, bulk soluble, bulk pellet from the STDC and UC process) were determined using the Advanced Protein Assay (Cytoskeleton, Denver, USA) according to the manufacturer’s description. Before the usage of the antigen preparation, endotoxin levels were determined using the Pierce Chromogenic Endotoxin Quant assay (ThermoFisher, Waltham, MA, USA). Only preparations below 0.1 EU/ml has been used for in vitro stimulation assays. Moreover, we performed in vitro stimulation using dendritic cells from naïve mice to confirm that the antigen preparations do not induce pro-inflammatory immune responses per se. To avoid multiple thawing and freezing, aliquots with sufficient antigen contents for each T-cell assay were pre-prepared and frozen at − 80 °C until required.

### SDS gel

A 10% SDS gel was prepared, and samples of different antigenic preparations and markers (MagicMark, ThermoFisher) were loaded on the gel and run for 45 min at 130 V. Afterwards, the gel was washed twice with distilled water for 5 min and stained with Coomassie brilliant blue (Sigma-Aldrich GmbH, Taufkirchen, Germany) for 30 min. Then, the gel was incubated for 20 min in 30% methanol (Merck, Darmstadt, Germany). Finally, methanol was exchanged, and the gel was incubated overnight (Supplementary Fig. 1).

### Parasitological assessment

Adult worms were recovered from the TC of individually infected BALB/c mice at two different time points, at the pre-patent (days 42 and 43 p.i.) and patent stage (days 71 and 72 p.i.). Hence, the TC of all mice was flushed with sterile PBS, expulsed worms were retained by passing through gauze and stored in 4% formalin, and the remaining fluid was stored at − 20 °C for further usage. Worm numbers and gender were determined microscopically as described earlier (Hübner et al. 2009b; Rodrigo et al. 2016; Torrero et al. 2010; Volkmann et al. 2003). In addition, worms were analyzed for length and developmental stages via microscopy. To determine the presence of Mf, 50 µl EDTA-treated peripheral blood and also 50 µl of thoracic cavity fluid were stained separately by using 300 µl of Hinkelmann’s solution and examined for the number of Mf (Al-Qaoud et al. 1997; Layland et al. 2015; Rodrigo et al. 2016).

### Determination of the immune cell composition within the TC lavage

Cytospins were prepared to analyze the composition of infiltrating immune cells within the TC lavage as described before (Ritter et al. 2017; Wiszniewsky et al. 2019). In short, glass slides were covered with Shandon filter cards and EZ Cytofunnels® and placed into Shandon Cytoclips (ThermoFisher Scientific, Schwerte, Germany). Then, 5 × 10^4^ TC cells were added and pulse centrifuged at 340 g without brake. Cytospin slides were dried overnight at room temperature and stained with a Diff-Quik staining set (Medion Diagnostics, Miami, USA) according to the manufacturer’s instructions, and 200 cells were microscopically differentiated into macrophages, eosinophils, neutrophils, and lymphocytes in a blinded manner to determine the frequency and percentage of each cell type.

### Preparation of mediastinal lymph nodes and dendritic cells

The draining mediastinal lymph nodes (mLN) were removed with a fine pair of sterile tweezers and placed in ice-cold PBS. Afterwards, isolated lymph nodes were squashed between round-ended tweezers, and resulting cell suspensions were transferred into 15 ml falcons. The mLN were washed with PBS for 5 min at 340 g (4 °C), re-suspended in PBS, and counted by microscopy. Bone marrow–derived dendritic cells (BMDCs) were generated as previously described (Ritter et al. 2010). In brief, recovered bone marrow samples from naïve BALB/c mice were washed, depleted of erythrocytes, and then differentiated over 7 days in Iscove’s modified Dulbecco’s medium supplemented with 10% FCS, 1% penicillin/streptomycin, 1% L-glutamine, 1% non-essential amino acids, 1% sodium pyruvate, 1% sodium bicarbonate (all from PAA), and 10 μg/ml granulocyte–macrophage colony-stimulating factor (GM-CSF; PeproTech, Rocky Hill, NJ, USA). After 7 days of generation, cultured BMDCs were harvested and re-suspended in RPMI 1640 medium containing 10% FCS, 1% penicillin/streptomycin, 1% L-glutamine, and 0.1% gentamycin.

### CD4^+^ T cell co-culture assays

BMDCs from naïve mice were plated in 96-well round-bottom plates at a concentration of 5 × 10^4^ cells/well in RPMI medium and left to settle for 2 h. Splenic CD4^+^ T cells from different time points of *L. sigmodontis* infection were freshly isolated using CD4^+^ beads (mouse CD4 [L3T4] MicroBeads) and the autoMACS Pro (both Miltenyi Biotec GmbH, Bergisch Gladbach, Germany) according to the manufacturer’s protocol, and purity was checked via flow cytometric analysis. CD4^+^ T cells were added to the BMDC cultures at a concentration of 2.5 × 10^5^ cells/well in RPMI medium and left either un-stimulated or activated with one of the different LsAg preparations (40 μg/ml). After 72 h, all cell culture supernatants were removed and analyzed for cytokines.

### Determination of cytokines and chemokines

Cytokine concentrations in the culture supernatants of T cell assays or the TC lavage were determined by ELISA according to the manufacturer’s instructions (TARC/CCL17, MDC/CCL22, eotaxin-1/CCL11, RANTES/CCL5, IP-10/CXCL10, MIP-2/CXCL2, granzyme B, TGF-β, IL-5, R&D Systems, Wiesbaden-Nordenstadt, Germany; IL-5, IL-13, IFN-γ, IL-10, BD Biosciences, Heidelberg, Germany). The assay ranges were as follows: CCL17 31.2–2000 pg/ml, CCL22 7.8–500 pg/ml, eotaxin-1 7.8–500 pg/m, RANTES 31.2–2000 pg/ml, IP-10 62.5–4000 pg/ml, MIP-2 15.6–1000 pg/ml, granzyme B 62.5–4000 pg/ml, TGF-β 31.2–2000 pg/ml, IL-5 15.6–1000 pg/ml, IFN-γ 3.2–200 pg/ml, and IL-10 31.3–2000 pg/ml. ELISA plates were read and analyzed at 450 and 570 nm using a SpectraMax 340PC384 photometer and SOFTmax PRO 3.0 software (Molecular Devices, Sunnyvale, CA).

### Statistical analysis

Statistical differences were determined using GraphPad PRISM 9.4.1 software (San Diego, CA). Data were tested for Gaussian distribution with the Kolmogorov–Smirnov, D’Agostino and Pearson omnibus normality, and Shapiro normality tests. Non-parametrically distributed data were tested using the Kruskal–Wallis tests followed by Dunn’s multiple comparison tests for further comparison of the groups, and *p*-values of 0.05 or less were considered statistically significant. For comparisons of continuous parameters, the Spearman correlation test was used.

## Results

### Increased worm length in patently infected mice

**Fig. 1:**
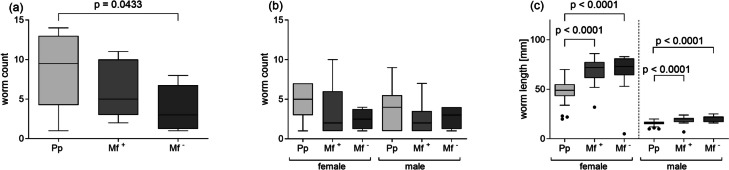
Higher numbers of adult parasites but shorter worms in infected pre-patent *L. sigmodontis*–infected BALB/c mice. **a** Worms were isolated from the thoracic cavity of individually *L. sigmodontis*–infected mice at the pre-patent (Pp) stage (days 42 and 43 p.i., *n* = 14) or the patent stage (days 71 and 72 p.i.), the latter one sub-grouped into Mf + (*n* = 11) or Mf − (*n* = 8) mice. **b** Absolute number of worms separated according to their gender at the pre-patent stage (*n* = 14) and in Mf + (*n* = 11) or Mf − (*n* = 8) within the patent stage. **c** Comparison of worm length separated according to their gender at the pre-patent stage (*n* = 14) and at the patent stage (Mf + (*n* = 11) or Mf − (*n* = 8)). Graphs show box whiskers with median, interquartile ranges, and outliers of data from individual mice of two independent infection studies. Statistical significances between the indicated groups were obtained after a Kruskal–Wallis followed by Dunn’s multiple comparison test for further comparison of the groups

Following simultaneous infection, parasite burden was measured after isolating worms from the TC of individual wildtype BALB/c mice infected with *L. sigmodontis* at either the pre-patent stage (days 42 and 43 p.i.) or the patent stage (days 71 and 72 p.i.). Mice within the patent group were further subdivided according to their Mf status. Pre-patent had a significantly higher worm burden than Mf** − **mice, but there was no significant difference between the Mf + and Mf − animals (Fig. 1a). Worm counts and the number of Mf from 71/72 days infected mice did not correlate (blood-derived Mf *r* = 0.339, *p* = 0.156; TC-derived Mf *r* = 0.155, *p* = 0.528). No significant differences in the number of male and female worms were observed between any of the groups (Fig. 1b). As expected, female worms were significantly longer than male worms at both time points, and the average worm length of female and male worms significantly increased at the patent stage when compared to the pre-patent stage (Fig. 1c). Female and male worm lengths in Mf + and Mf − mice showed no differences (Fig. 1c).

### Eosinophil counts are highest during pre-patent and microfilariae-positive infections

**Fig. 2:**
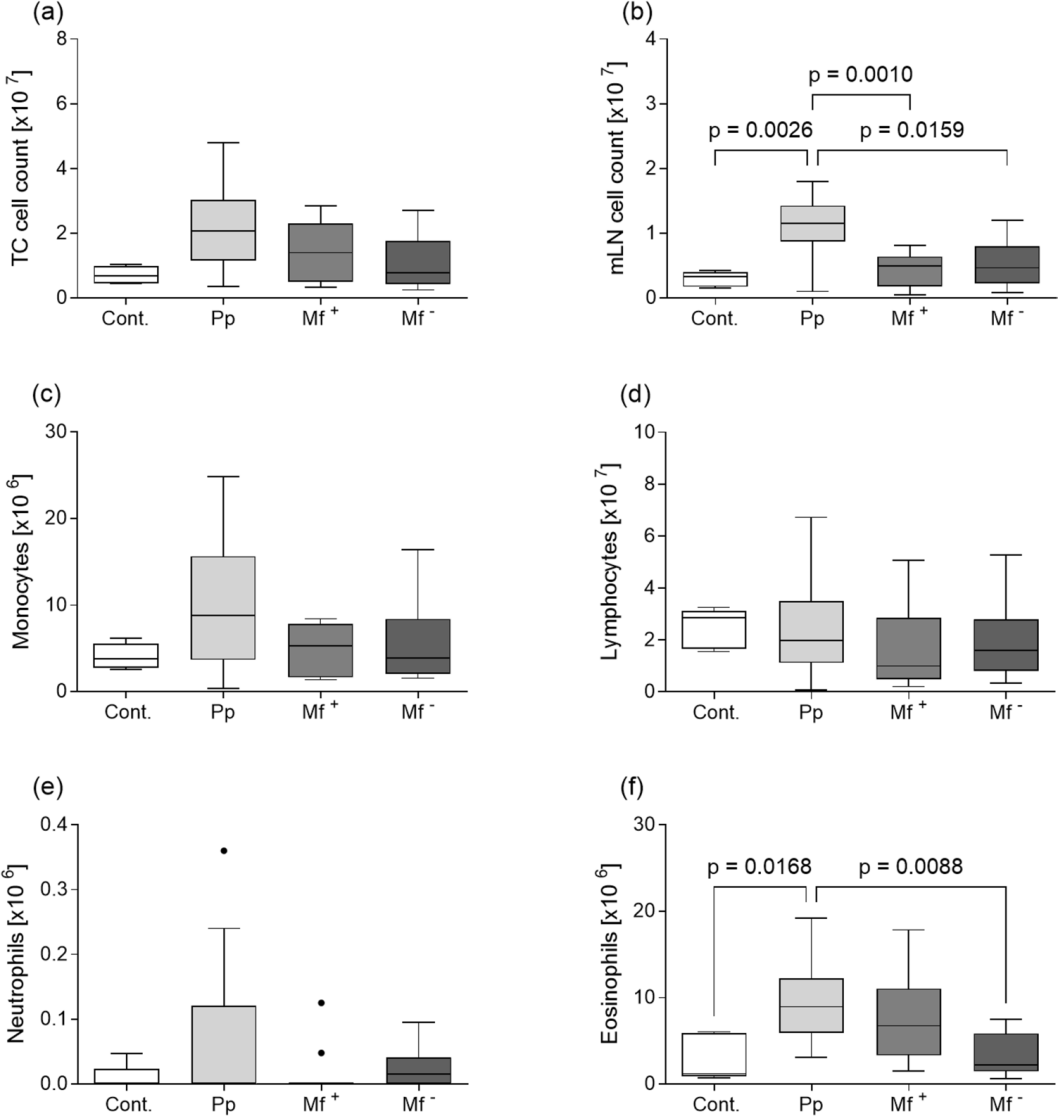
Adult worm infections present expanded eosinophil populations at the site of infection. Total cell numbers within **a** the thoracic cavity (TC) and **b** the mediastinal lymph nodes (mLN) of uninfected (Cont.) and individually *L. sigmodontis*–infected mice at the pre-patent (Pp) stage (days 42 and 43 p.i.) or the patent stage (days 71 and 72 p.i.) subdivided into Mf + and Mf − . Absolute cell numbers of monocytes (**c**), lymphocytes (**d**), neutrophils (**e**), and eosinophils (**f**) in the TC. Box plots show cell counts in the described tissues of the control group containing non-infected mice (*n* = 5), pre-patent (*n* = 18), Mf + (*n* = 11), and Mf − (*n* = 8) infected mice. Graphs show box whiskers with median, interquartile ranges, and outliers of data from individual mice of two independent infection studies. Statistical significances between the indicated groups were obtained after a Kruskal–Wallis followed by Dunn’s multiple comparison test for further comparison of the groups

To assess the effects of *L. sigmodontis* on cellular influx, total cell numbers in the TC and mLN were determined. When compared to uninfected (Cont.), Mf + , and Mf − mice, the absolute cell number in the TC and mLN in pre-patent mice was increased, albeit only statistically significant in the mLN (Fig. 2a, b respectively). No differences were observed between Mf + and Mf − infected mice. In addition, TC cells were collected and stained to determine the absolute number of monocytes, lymphocytes, neutrophils, and eosinophils. As depicted in Fig. 2c, no significant differences were observed between the groups in terms of monocytes, although there was a slight increase in the pre-patent mice. The numbers of lymphocytes and neutrophils did not significantly differ among the groups (Fig. 2d, e). Interestingly, eosinophil numbers were significantly higher in pre-patent mice when compared to both uninfected and Mf − mice (Fig. 2f). Although not statistically significant, eosinophil numbers were higher in Mf + mice compared to the Mf − ones, and TC-derived Mf counts did significantly correlate to the number of eosinophils (*r* = 0.717, *p* = 0.001).

### Granzyme B and eotaxin-1 are elevated in pre-patent mice

**Fig. 3:**
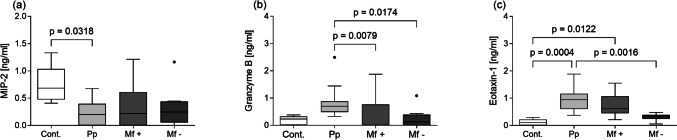
Increased levels of eotaxin-1 and granzyme B in the pre-patent phase. Levels of MIP-2 (**a**), granzyme B (**b**), and eotaxin-1 (**c**) were measured within the thoracic cavity of naïve mice (Cont.) (*n* = 5), infected mice at the pre-patent (Pp) (*n* = 18), and patent stages subdivided into Mf + (*n* = 11) and Mf − (*n* = 8) mice. Graphs show box whiskers with median, interquartile ranges, and outliers of data from individual mice of two independent infection studies. Statistical significances between the indicated groups were obtained after a Kruskal–Wallis followed by Dunn’s multiple comparison test for further comparison of the groups

The crucial role of cytokines within the TC during the infection with *L. sigmodontis* has already been shown (Frohberger et al. 2019; Reichwald et al. 2022). Alongside cytokines, chemokines are also important mediators of host immune responses as they induce the recruitment of cells to the site of infection. Thus, we measured a panel of chemokines in the TC lavage (Fig. 3). With regards to RANTES, IP-10, CCL17, and CCL22, we did not observe any significant differences between the groups (Supplementary Fig. 2). However, levels of MIP-2 were lower in infected mice, albeit statistically significant differences were only observed between pre-patent and control animals (Fig. 3a). In contrast, granzyme B levels were highest in the pre-patent mice, and this difference was statistically significant when compared to both Mf + and Mf − mice (Fig. 3b). Granzyme B also correlated positively with the number of eosinophils, monocytes, and lymphocytes, all TC and mLN cells (Table S1). Eotaxin-1 was the only chemokine to remain elevated throughout infection when compared to uninfected mice (Fig. 3c), following a similar pattern as eosinophil numbers (Fig. 2f). Eotaxin-1 levels correlated with the number of Mf (blood-derived Mf *r* = 0.615, *p* = 0.005; TC-derived Mf *r* = 0.544, *p* = 0.016).

### Increased IL-13 levels in re-stimulated CD4^+^ T cells of patently *L. sigmodontis*–infected BALB/c mice is independent of the Mf status

Next, we aimed to determine whether filarial-specific T cell responses differed between pre-patent and patent phases and, moreover, whether responses were stronger with varying worm antigen preparations. In short, CD4^+^ splenic T cells from pre-patent and Mf + or Mf − *L. sigmodontis–*infected BALB/c mice or controls were co-cultured with BMDCs from naïve BALB/c mice. These cells were left either un-stimulated or activated with the different *L. sigmodontis* antigen (from total, female, or male worms) preparations (STDC or UC) for 72 h.

**Fig. 4:**
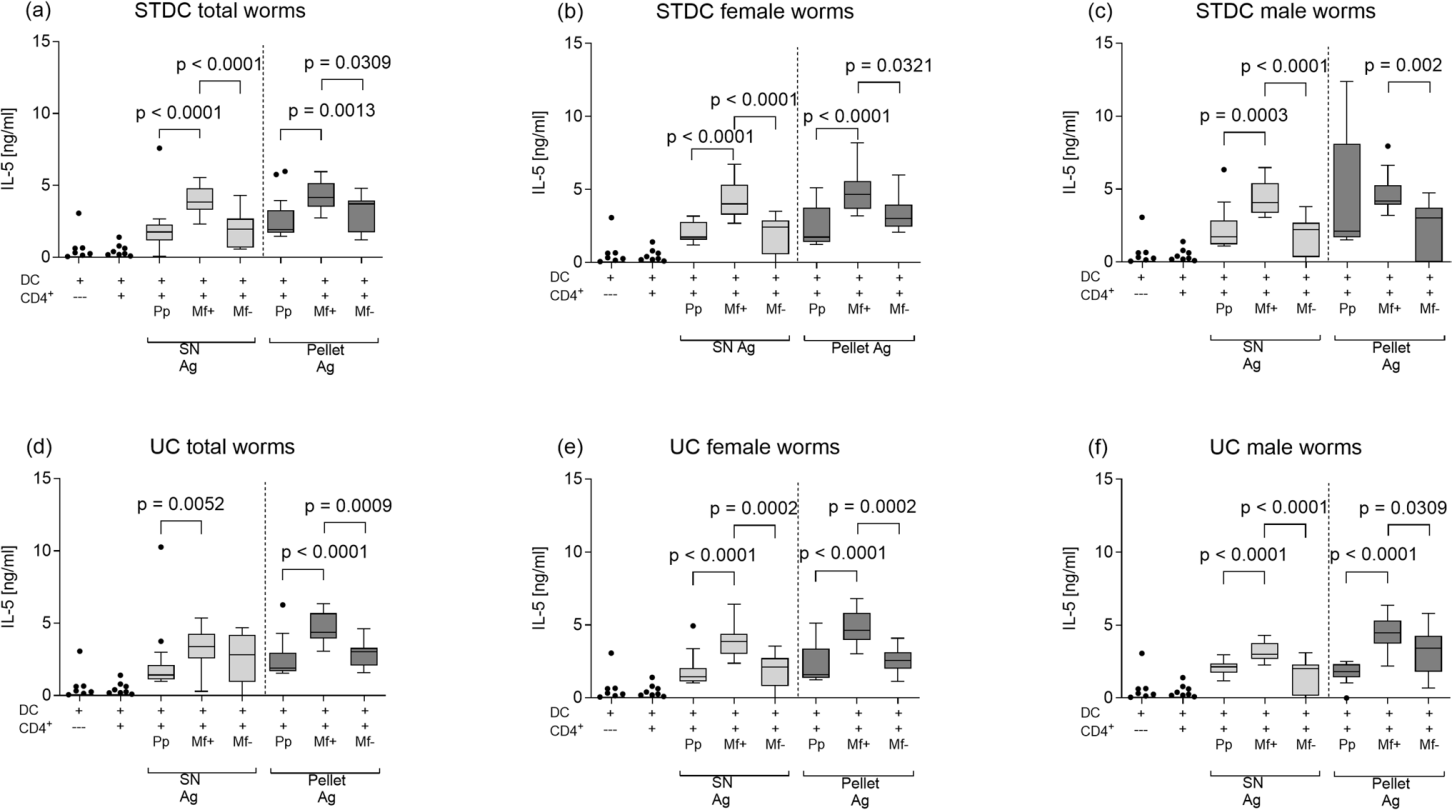
Increased IL-5 responses in patently infected Mf + BALB/c mice. Wildtype BALB/c mice were naturally infected with *L. sigmodontis*, and the IL-5 production of ex vivo re-stimulated splenic CD4^+^ T cells from Pp, Mf + , and Mf − mice was determined: CD4^+^ T cells were co-cultured for 72 h with BMDCs and left either un-stimulated or activated with different LsAg (total, female, male worms) preparations, thus, total (**a**), female (**b**), and male (**c**) LsAg prepared by standard centrifugation (STDC) and total (**d**), female (**e**), and male (**f**) prepared by ultracentrifugation (UC). From two independent infection studies, graphs show box whiskers with median, interquartile ranges, and outliers of three assays at the pre-patent stage, four assays of Mf + , and three assays of Mf − using isolated CD4^+^ T cells pooled from 4 or 5 mice per group. Statistical significances between the indicated groups were obtained after a Kruskal–Wallis followed by Dunn’s multiple comparison test for further comparison of the groups (**a**–**f**)

The overall picture of IL-5 release was comparable regardless of the used centrifugation method and independent of the used fraction (pellet or SN) since, in all assays, cells from Mf + mice produced more IL-5 than cells from the pre-patent stage (Fig. 4a–f). Furthermore, when comparing the IL-5 production in T cell cultures from Mf + and Mf − mice, significantly less IL-5 was released from cells of Mf − mice in all settings (Fig. 4a–c, e–f) apart from “UC total worms” (Fig. 4d). In general, similar responses were seen with the pellet preparation and the soluble SN fraction (Fig. 4a–f).

**Fig. 5:**
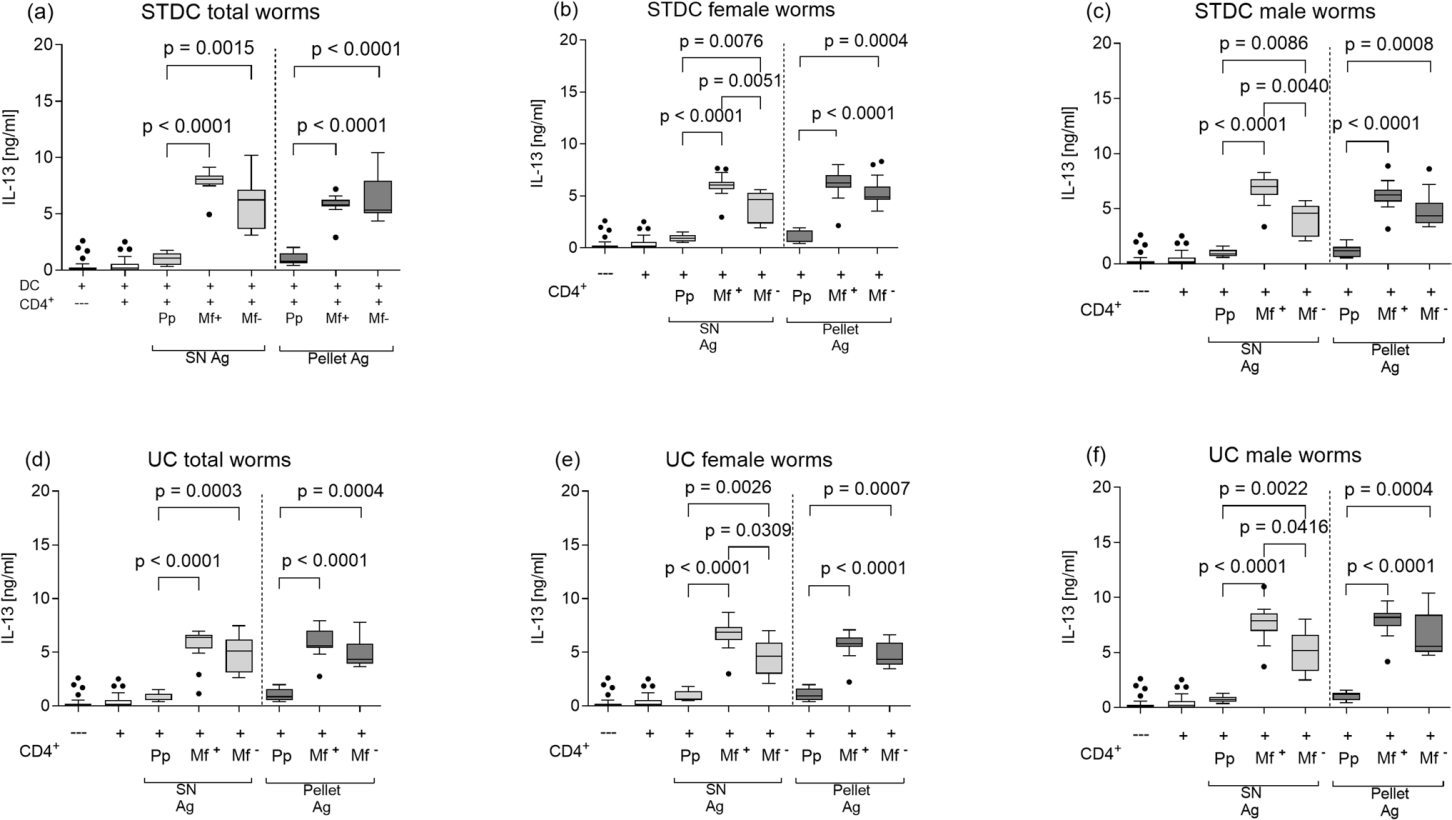
Elevated filarial-specific IL-13 responses in the chronic phase of the infection. Wildtype BALB/c mice were naturally infected with *L. sigmodontis*, and the IL-13 production of ex vivo re-stimulated splenic CD4^+^ T cells from pre-patent, Mf + , and Mf − mice was determined: CD4^+^ T cells were co-cultured for 72 h with BMDCs and left either un-stimulated or activated with different LsAg (total, female, male worms) preparations, thus, total (**a**), female (**b**), and male (**c**) LsAg prepared by standard centrifugation (STDC) and total (**d**), female (**e**), and male (**f**) prepared by ultracentrifugation (UC). Graphs show box whiskers with median, interquartile ranges, and outliers of three assays at the pre-patent stage, four assays of Mf + , and three assays of Mf − using isolated CD4^+^ T cells pooled from 4 or 5 mice per group from two independent infection studies. Statistical significances between the indicated groups were obtained after a Kruskal–Wallis followed by Dunn’s multiple comparison test for further comparison of the groups (**a**–**f**)

In contrast to the above-mentioned IL-5, we observed a general upregulation in IL-13 secretion in cultures from both Mf + mice and Mf − animals, and this was independent of the centrifugation method and the use of SN or pellet-derived antigen extracts (Fig. 5a–f). T cells from pre-patent mice produced levels of IL-13 comparable to naïve mice (Fig. 5a–f) regardless of the antigen source. Moreover, comparisons of CD4^+^ T cell responses from Mf + and Mf − mice revealed statistically significant higher levels of IL-13 by Mf + -derived cells with soluble but not pellet-derived female or male worms (Fig. 5b, c, e, f). Despite this, IL-13 levels were comparable between pellet and soluble antigen preparations within the same groups of mice (Fig. 5a–f).

### IFN-γ and IL-10 responses increase in patently infected mice

**Fig. 6:**
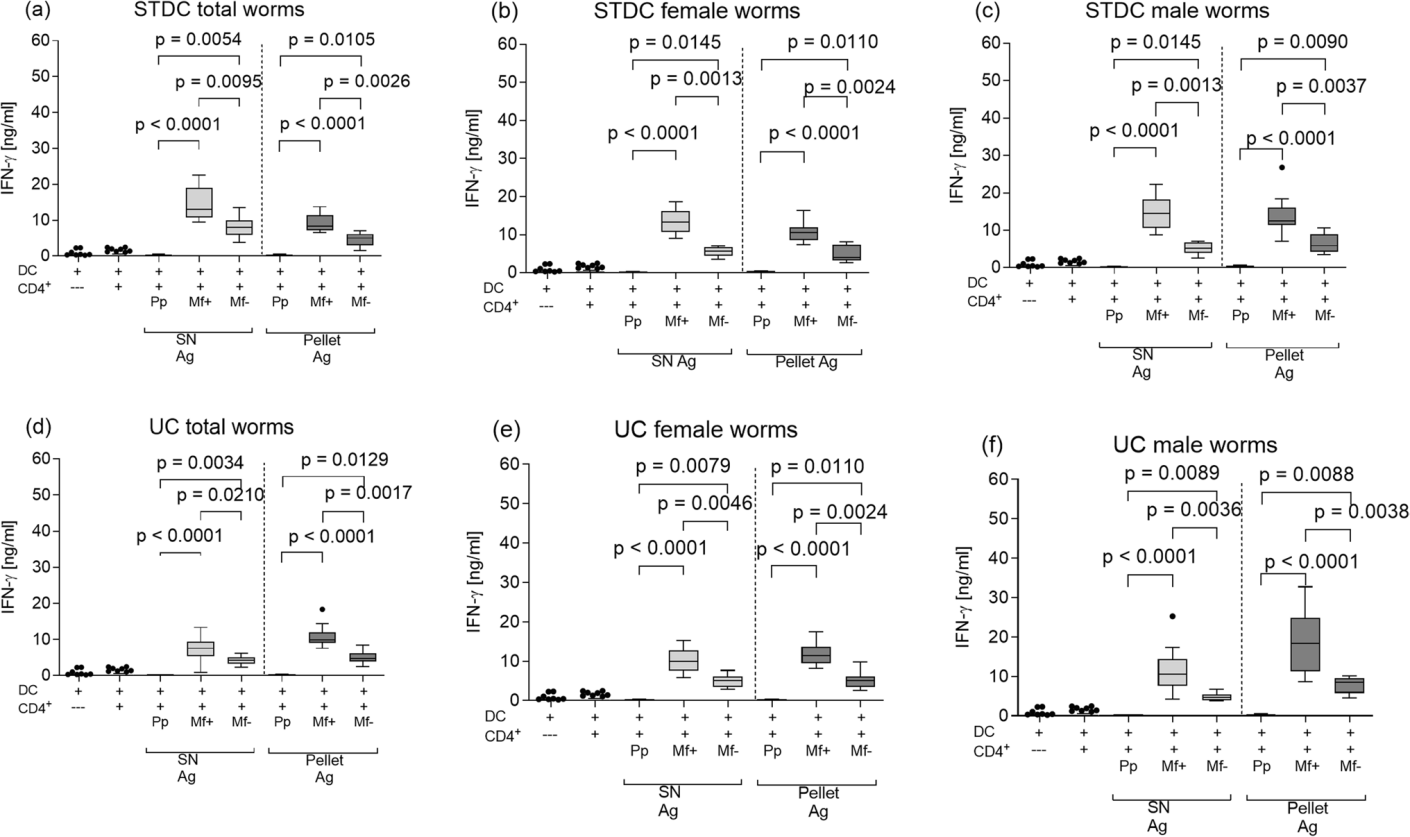
Altered filarial-specific IFN-γ during pre-patency and patency. Wildtype BALB/c mice were naturally infected with *L. sigmodontis*, and the IFN-γ production of ex vivo re-stimulated splenic CD4^+^ T cells from pre-patent, Mf + , and Mf − mice was determined: CD4^+^ T cells were co-cultured for 72 h with BMDCs and left either un-stimulated or activated with different LsAg (total, female, male worms) preparations, thus, total (**a**), female (**b**), and male (**c**) LsAg prepared by standard centrifugation (STDC) and total (**d**), female (**e**), and male (**f**) prepared by ultracentrifugation (UC). Graphs show box whiskers with median, interquartile ranges, and outliers of three assays at the pre-patent stage, four assays of Mf + , and three assays of Mf − using isolated CD4^+^ T cells pooled from 4 or 5 mice per group from two independent infection studies. Statistical significances between the indicated groups were obtained after a Kruskal–Wallis followed by Dunn’s multiple comparison test for further comparison of the groups (**a**–**f**)

As synergistic effects of Th2 and Th1 cytokines during murine filariasis have been previously described (Saeftel et al. 2003), we determined whether there were any differences in the release of IFN-γ from the ex vivo re-stimulated CD4^+^ T cells at the specific stages of infection. As shown in Fig. 6, IFN-γ levels were significantly higher in cultures from the chronic infection stage, whereas no IFN-γ was produced in cultures from cells at the pre-patent phases regardless of which LsAg fractions or centrifugation processes were used (Fig. 6a–f). Moreover, IFN-γ production was significantly lower in co-cultures of Mf − mice when compared to those from Mf + mice (Fig. 6a–f). When comparing responses of soluble and pellet-derived antigens, levels of IFN-γ productions were comparable between both antigenic preparations. In general, the stimulation with the standard centrifugation-based antigenic extract yielded slightly higher IFN-γ levels.

**Fig. 7:**
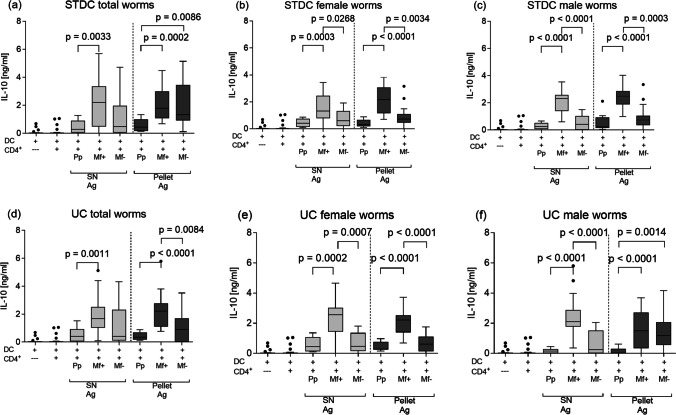
Filarial-specific IL-10 responses by CD4^+^ T cells from infected BALB/c mice at the pre-patent and patent stage. Wildtype BALB/c mice were naturally infected with *L. sigmodontis*, and the IL-10 production of ex vivo re-stimulated splenic CD4^+^ T cells from pre-patent, Mf + , and Mf − mice was determined: CD4^+^ T cells were co-cultured for 72 h with BMDCs and left either un-stimulated or activated with different LsAg (total, female, male worms) preparations, thus, total (**a**), female (**b**), and male (**c**) LsAg prepared by standard centrifugation (STDC) and total (**d**), female (**e**), and male (**f**) prepared by ultracentrifugation (UC). Graphs show box whiskers with median, interquartile ranges, and outliers of three assays at the pre-patent stage, four assays of Mf + , and three assays of Mf − using isolated CD4^+^ T cells pooled from 4 or 5 mice per group from two independent infection studies. Statistical significances between the indicated groups were obtained after a Kruskal–Wallis followed by Dunn’s multiple comparison test for further comparison of the groups (**a**–**f**)

Next, we measured IL-10 in the same in vitro experimental setup. Lower concentrations of IL-10 were detected in cultures from pre-patent mice regardless of the source of antigen preparation (Fig. 7a–f). All soluble extracts from both standard and UC processes elicited stronger IL-10 release from CD4^+^ T cells from Mf + mice compared to cultures of pre-patent mice (Fig. 7a–f) and also partly to cultures from Mf − mice (Fig. 7b, c, e, f). Interestingly, IL-10 release was equal in T cell assays from Mf + and Mf − mice using the pellet-derived “STDC total worms” and “UC male worms” antigens (Fig. 7a, f, respectively).

Collectively, these data show that re-stimulated cells from Mf + mice responded strongest to presented filarial antigenic preparations, regardless of its worm composition or the applied centrifugation process. Moreover, the data show that CD4^+^ T cells in the pre-patent phase are “quiet” since, except for IL-5, neither IL-13, IL-10, nor IFN-γ were detected in significant concentrations.

## Discussion

One possible limitation to comparing parasite-specific immune responses from different studies is the usage of different protocols for the preparation of parasite antigens and different in vitro culture settings (Anuradha et al. 2016; Arndts et al. 2012; Arndts et al. 2014; Babu et al. 2009; Babu et al. 2005; Gentil et al. 2014; Ritter et al. 2017; Rodrigo et al. 2016; Specht et al. 2011; Ziewer et al. 2012). When analyzing filarial-specific responses, it is important, if possible, to use the antigen from the infecting filarial species as differences may arise. Accordingly, comparisons of cell cultures from *O. volvulus–*infected individuals presented different responses to *O. volvulus–* and *Brugia malayi*–derived antigens (Arndts et al. 2014). Nevertheless, it is rarely taken into consideration whether T cell responses are affected when extracts stem from male, female, or mixed-sex filariae. Moreover, even though studies have explored the immune modulating effects of distinct molecules (e.g., cystatins, ES-62) (Al-Riyami and Harnett 2012; Hartmann et al. 2002; Lumb et al. 2019), for the preparation of total antigenic material, there are currently no standardized protocols, which could affect the composition of lipids, glycoproteins, and proteins. Therefore, this study compared the cytokine release from CD4^+^ T cells isolated from *L. sigmodontis*–infected mice to soluble or pellet extracts prepared from male, female, or total *L. sigmodontis* worms. In addition, soluble preparations were obtained via standard centrifugation and ultracentrifugation, albeit neither would have removed exosomes. In general, the composition of the different preparations was slightly different according to SDS electrophoresis results (Supplementary Fig. 1). However, cytokine profiles of immune cells derived from *L. sigmodontis*–infected mice were comparable upon stimulation with the different preparations, showing that despite the differences in the composition, the antigenic preparations lead to comparable immune responses in vitro. With all measured parameters, except IL-5, cytokine responses were low to non-detectable in cell cultures with CD4^+^ T cells from pre-patent mice. This is somewhat puzzling since, at day 30, worms are in an adult stage and Mf production starts from day 50. However, it was already shown that TC-derived lymph node cells from *L. sigmodontis*–infected BALB/c mice produced lower IL-5 levels at the pre-patent time points when stimulated with LsAg when compared to cells from the patent time point (Taylor et al. 2005). A previous study with *L. sigmodontis* also described that the systemic type 2 immune response shows a peak 5 to 6 weeks after infection, just prior to the onset of microfilaremia (Boyd et al. 2015). Moreover, we observed that cultures from latent mice on day 72 were significantly lower than those from patent mice including IFN-γ, which expands on our earlier findings (Rodrigo et al. 2016). Thus, it appears that an increase in cytokine release might be a result of an activation of T cells induced by the previous contact with circulating Mf. In line with this, earlier in vivo data showed that the injection of Mf induces the production of IFN-γ and other pro-inflammatory mediators (Hübner et al. 2008). We hypothesize that distinct immune parameters dampen CD4^+^ T cell responses which are required to prevent worm development and survival in BALB/c mice, e.g., via the polarization into Th17 T cells (Taylor et al. 2005; Wiszniewsky et al. 2021).

Immunologically, filariae are unique in their requirement for *Wolbachia* endosymbionts. All of our extract preparations contained *Wolbachia*, whereas only the total and the female worm extracts included both *Wolbachia* and Mf components. Thus, we had anticipated differences in cytokine release by CD4^+^ T cells upon stimulation in comparison to *Wolbachia*-containing but Mf-lacking male worm antigen. Especially since Mf-exposed monocyte-derived human DCs demonstrated marked mRNA expression of toll-like receptors and upregulation of diverse chemokines in previous studies (Semnani et al. 2011, 2008), stimulation of monocytes from non-endemic donors with Mf lysate directly inhibited CD4^+^ T cell proliferation and cytokine production (O’Regan et al. 2014). Next to the comparison of pre-patent, Mf + , and Mf − infections, we assessed the impact of different adult worm extract preparations on the re-stimulation of T cells from naïve, pre-patent, Mf + , and Mf − animals. Usually, worm antigen is prepared after homogenizing adult worms of both genders followed by a short centrifugation (Ajendra et al. 2014). Therefore, supernatants from this preparation method may contain non-soluble particles, e.g., the cuticula including glycanes and chitohexaose, which were previously shown to modulate immune responses (Hübner et al. 2013; Panda et al. 2013; Prasanphanich et al. 2013; Ritter et al. 2010). However, here, in vitro immune responses were not altered due to the different worm extracts but rather by the time points of infection. In addition, the lack of modulation by pellet and soluble extracts showed that responses remained comparable in all antigen preparation scenarios. Thus, future studies can use a crudely prepared extract that does not require an additional ultracentrifugation process. Moreover, these data are also important for discovering the potential immunomodulatory capacities of LsAg for autoimmune and metabolic disorders (Ajendra et al. 2016; Berbudi et al. 2016; Hübner et al. 2009a).

As mentioned earlier, the *L. sigmodontis* life cycle in BALB/c mice is fully permissive. Nevertheless, only 40–60% of infected mice show a patent state. Th2 responses are critical for controlling the development of *L. sigmodontis* worms: in their absence, a higher worm burden occurs as well as an extended microfilaremia (Al-Qaoud et al. 1997; Frohberger et al. 2019; Hübner et al. 2009a; Layland et al. 2015; Ritter et al. 2017), and the same inverse correlation was shown with regards to eosinophils (Frohberger et al. 2019; Martin et al. 2000). Interestingly, both IL-4Rα/IL-5- and IFN-γ-deficient BALB/c mice are characterized by elevated parasite numbers and Mf which indicates the synergistic effects of Th1 and Th2 cytokines in controlling worm burden (Saeftel et al. 2003). Our previous studies also showed that both local and systemic cytokine release during in vitro co-culture assays with live Mf were lower in cells arising from Mf-negative mice (Rodrigo et al. 2016). In line, using the standard centrifugation protocol, the studies performed here revealed stronger responses of CD4^+^ T cells from patent mice than those observed in cultures from Mf-negative mice at the same stage of infection (day 72 p.i.). The present study further revealed that this effect was consistently statistically significant using worm extracts from both sexes and included Th1 responses (IFN-γ) as well. With regards to IL-5, a hallmark of helminth infections, CD4^+^ T cell responses in pre-patent and Mf-negative mice were comparable which strongly indicates that without developing microfilaremia, responses are dampened or Mf trigger T cell responses. Nevertheless, T cell responses in pre-patent versus chronic mice were constantly lower.

In general, we did not detect major differences comparing the various antigen preparations; thus, it seemed not to play a role if the standard or the ultracentrifugation method was used. Similarly, we observed only a slightly modulated cytokine production for the pellet antigen when compared to its SN counterpart within the Mf + and Mf − mice. In addition, antigens prepared from either gender did not drastically alter the immune profiles of CD4^+^ T cells in the measured cytokines, despite the fact that previous studies indicated that *Wolbachia* are necessary to induce pro-inflammatory immune responses to antigens derived from *B. malayi* (Taylor et al. 2009; Turner et al. 2006) and female adult worms carry the highest load of these endosymbiotic bacteria (Hise et al. 2004).

The aim of this study was to distinguish CD4^+^ T cell responses in pre-patent and patent periods of infection to different preparations of filarial worm extracts. Expanding on our previous studies revealing that CD4^+^ T cells from Mf + mice produced stronger responses than those from Mf − mice, here, we show that this observation remains with (a) different antigen preparations and (b) different worm genders.

## Supplementary Information

Below is the link to the electronic supplementary material.

Supplementary Fig. 1 SDS-PAGE of bulk LsAg after Coomassie Brilliant Blue staining containing supernatent (SN) and pellet after centrifugation of the antigen solution for 10 minutes at 1,485 g (STDC) or 2 hours with 50,000 g (UC). (PNG 118 kb)

High resolution image (TIF 182 KB)

Supplementary Fig. 2 Levels of RANTES, IP-10, CCL17 and CCL22 are not significantly altered in infected mice. Chemokine levels of RANTES (a), IP-10 (b), CCL17 (c) and CCL22 (d) were measured within the thoracic cavity of naïve mice (Cont.) (*n *=  5), infected mice at the pre-patent (*n *= 18) and patent stages subdivided into Mf+ (*n *= 11) and Mf- (*n *= 8) mice. Graphs show box whiskers with median, interquartile ranges and outliers of data from individual mice of two independent infection studies. Statistical significances were tested with Kruskal-Wallis test. (PNG 76 kb)

High resolution image (TIF 509 KB)

Supplementary file3 (XLSX 12.2 KB)

## Data Availability

The datasets used and/or analyzed during the current study are available from the corresponding author on reasonable request.

## Abbreviations

LsAg: *L. sigmodontis* Antigenic extracts
Ls: *Litomosoides sigmodontis*
Mf +: Microfilariae positive
Mf −: Microfilariae negative
Mf: Microfilariae
