# Supplementary figures and images for: Differences of in vitro immune responses between patent and pre-patent Litomosoides sigmodontis–infected mice are independent of the filarial antigenic stimulus used
Source: Parasitol Res. 2024 Oct 22;123(10):358. doi: 10.1007/s00436-024-08365-0 (PMC11496330; doi:10.1007/s00436-024-08365-0)

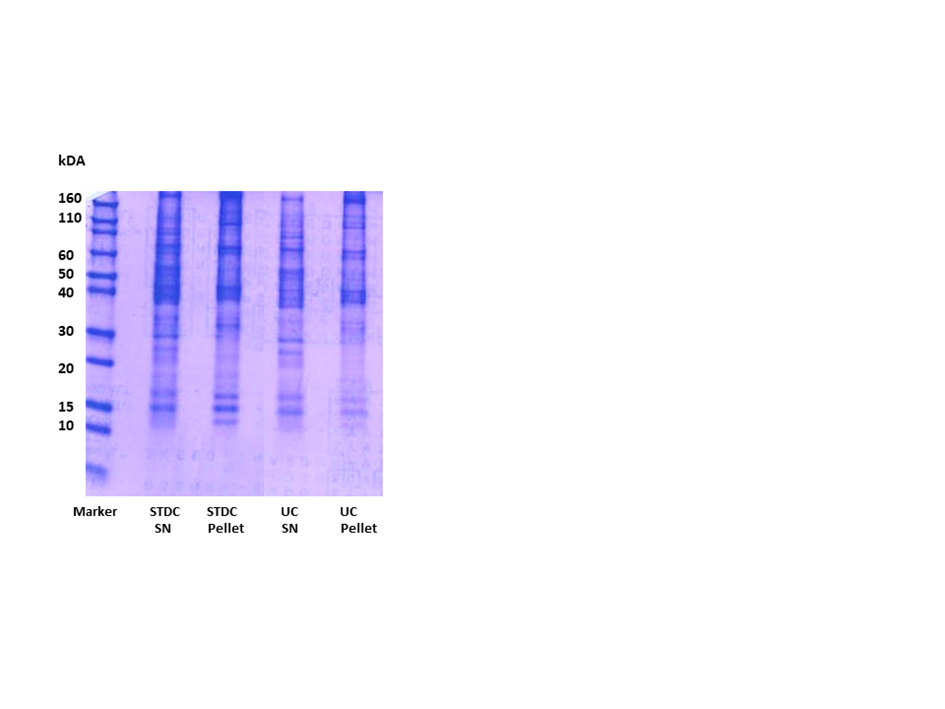

Supplement: Supplementary file 1 — SDS-PAGE of bulk LsAg after Coomassie Brilliant Blue staining containing supernatent (SN) and pellet after centrifugation of the antigen solution for 10 minutes at 1,485 g (STDC) or 2 hours with 50,000 g (UC). (PNG 118 kb) [file 436_2024_8365_Fig8_ESM.png]

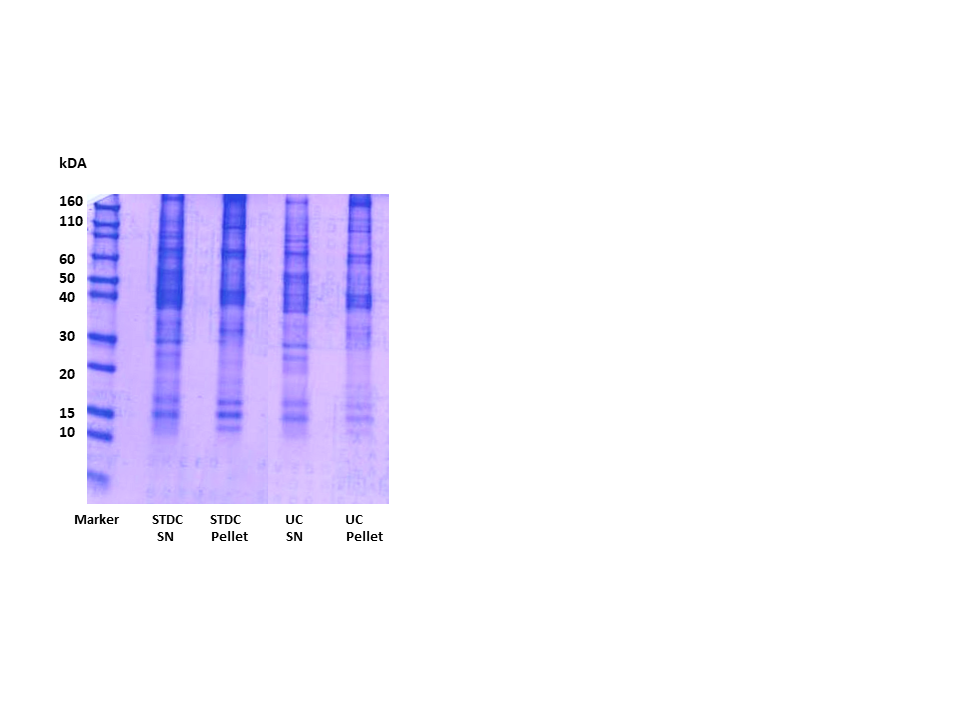

Supplement: Supplementary file 2 — High resolution image (TIF 182 KB) [file 436_2024_8365_MOESM1_ESM.tif]

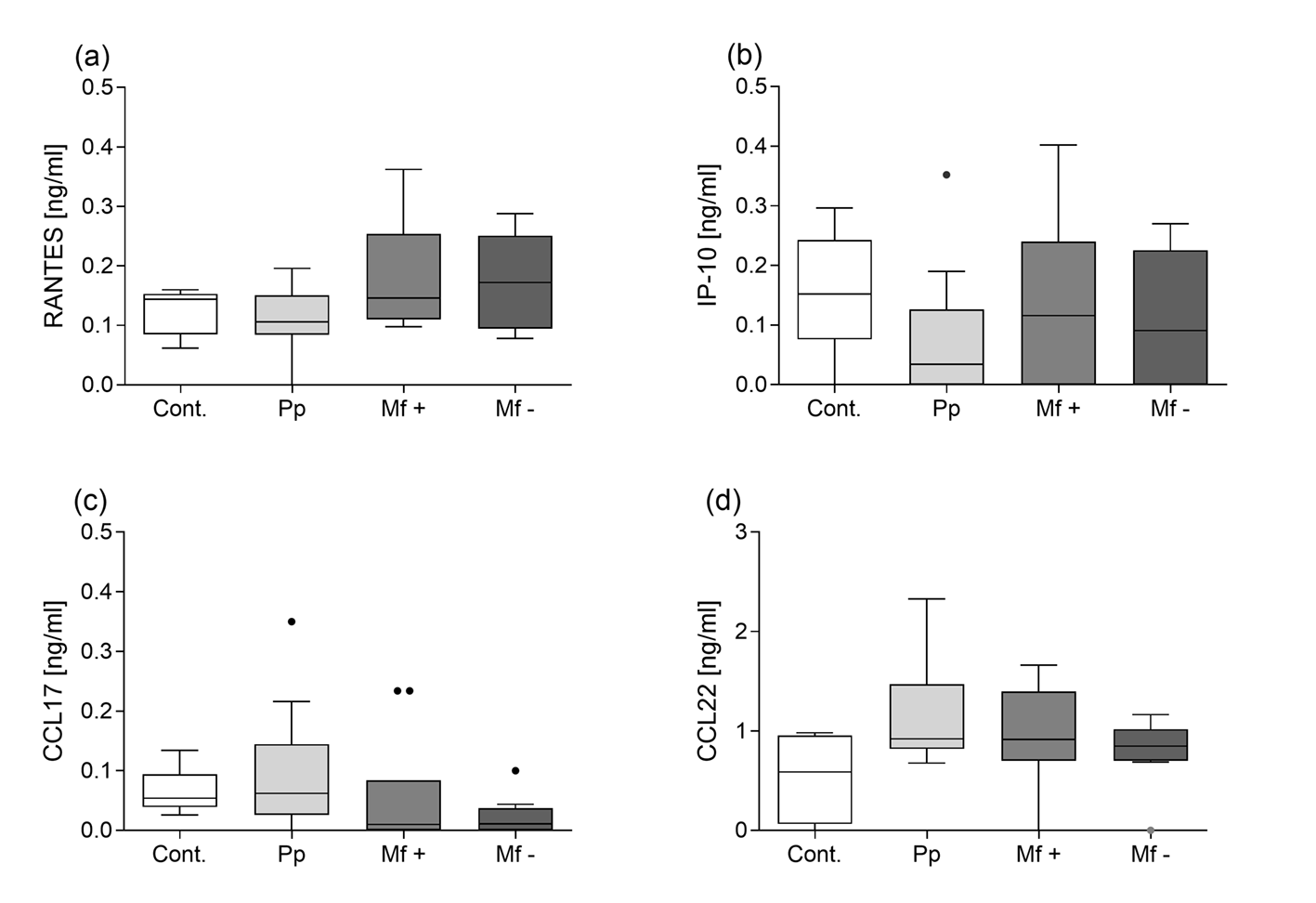

Supplement: Supplementary file 3 — Levels of RANTES, IP-10, CCL17 and CCL22 are not significantly altered in infected mice. Chemokine levels of RANTES (a), IP-10 (b), CCL17 (c) and CCL22 (d) were measured within the thoracic cavity of naïve mice (Cont.) (n = 5), infected mice at the pre-patent (n = 18) and patent stages subdivided into Mf+ (n = 11) and Mf- (n = 8) mice. Graphs show box whiskers with median, interquartile ranges and outliers of data from individual mice of two independent infection studies. Statistical significances were tested with Kruskal-Wallis test. (PNG 76 kb) [file 436_2024_8365_Fig9_ESM.png]

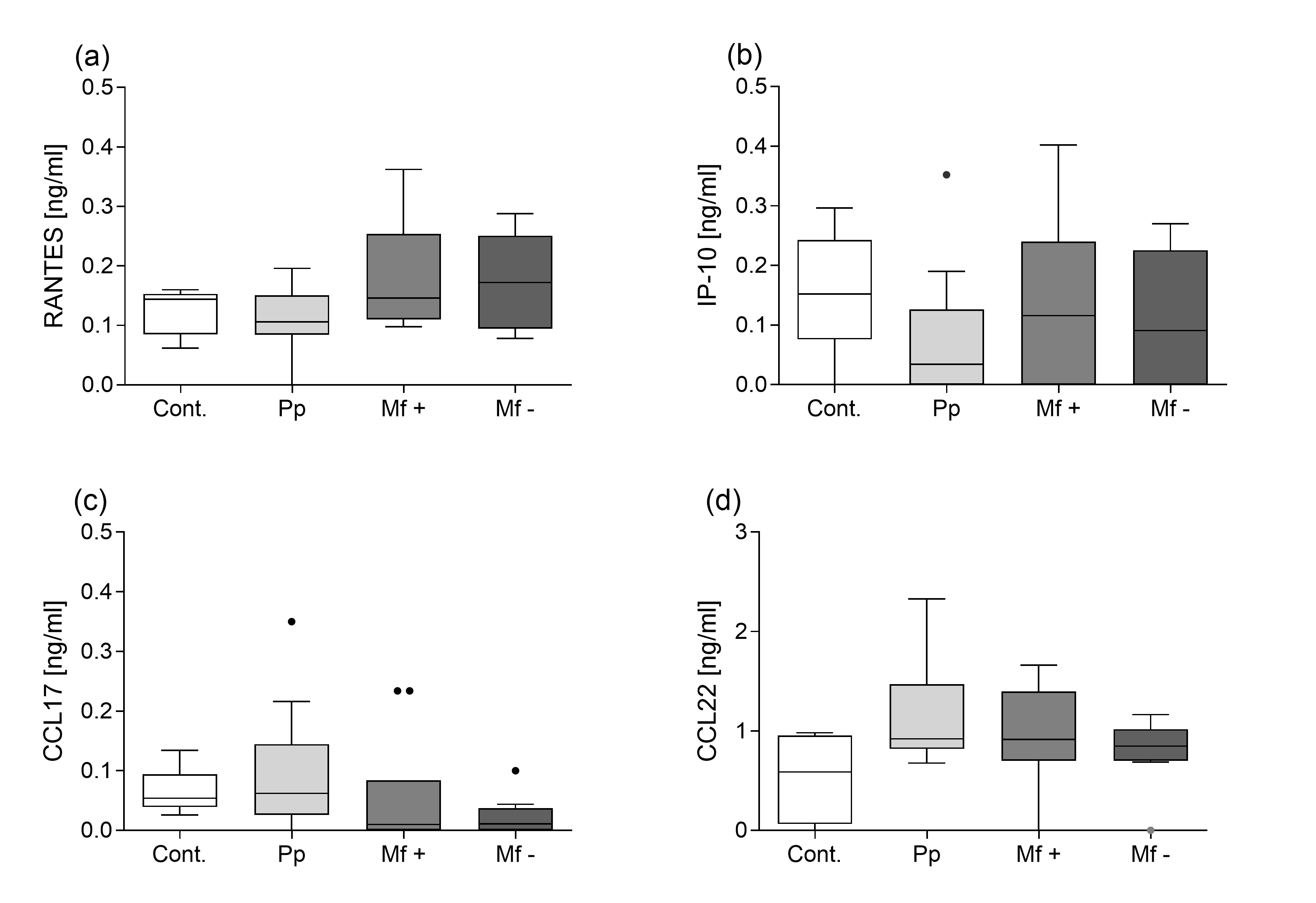

Supplement: Supplementary file 4 — High resolution image (TIF 509 KB) [file 436_2024_8365_MOESM2_ESM.tif]
